# pATM and γH2AX are effective radiation biomarkers in assessing the radiosensitivity of ^12^C^6+^ in human tumor cells

**DOI:** 10.1186/s12935-017-0419-5

**Published:** 2017-04-26

**Authors:** Jin Zhao, Zhong Guo, Shuyan Pei, Lei Song, Chenjing Wang, Jianxiu Ma, Long Jin, Yanqing Ma, Renke He, Jianbin Zhong, Ying Ma, Hong Zhang

**Affiliations:** 1 Medical College of Northwest Minzu University, Lanzhou, 730030 People’s Republic of China; 2 Department of Radiation Medicine, Institute of Modern Physics, Chinese Academy of Science, Lanzhou, 730030 People’s Republic of China

**Keywords:** ^12^C^6+^, Human tumor cells, Survival fraction, γH2AX, ATM

## Abstract

**Background:**

Tumour radiosensitivity would be particularly useful in optimizing the radiation dose during radiotherapy. The aim of the current study was to evaluate the potential value of phosphorylated H2AX (γH2AX) and ATM (pATM) in assessing ^12^C^6+^ radiosensitivity of tumour cells.

**Methods:**

Human cervical carcinoma HeLa cells, hepatoma HepG2 cells, and mucoepidermoid carcinoma MEC-1 cells were irradiated with different doses of ^12^C^6+^. The survival fraction was assayed with clonogenic survival method and the foci of γH2AX and pATM was visualized using immunocytochemical methods. Flow cytometry was used to assay γH2AX, pATM and the cell cycle.

**Results:**

The survival fraction decreased immediately in dose-dependent manner, but in turn, significantly increased during 24 h after ^12^C^6+^ irradiation. Both γH2AX and pATM foci accumulated linearly with doses and with a maximum induction at 0.5 h for γH2AX and 0.5 or 4 h for pATM, respectively, and a fraction foci kept for 24 h. The expression of γH2AX and pATM was in relation to cell cycle. The G0/G1 phase cells had the highest expression of γH2AX after 0.5 h irradiation and then decreased to a lower level at 24 h after irradiation. An obvious increase of pATM in G2/M phase was shown after 24 h of 2 and 4 Gy irradiation. The significant G2/M phase arrest was shown. There was a close relationship between the clonogenic survival and γH2AX and pATM expression both in timing and dose in response to ^12^C^6+^.

**Conclusions:**

The rate of γH2AX and pATM formation and loss may be an important factor in the response of cells to ^12^C^6+^. pATM and γH2AX are effective radiation biomarkers in assessing the radiosensitivity of ^12^C^6+^ in human tumor cells.

## Background

Radiation-induced cell death is mediated through induction of double-strand breaks (DSB) in DNA, which are lethal to cells if not repaired [1]. The energy deposition by low-linear energy transfer (LET) radiation is distributed randomly throughout the cell, whereas the energy from high- linear energy transfer (LET) radiation is deposited as discrete tracks where the particle has passed through the cell [2]. As a result, the DNA damage induced by high-LET heavy ion radiation is more complex than that by X- or gamma rays and leads to more severe biological consequences [3]. Although these effects can lead to cell death, mutations, genomic instability, or carcinogenesis, problems associated with the repair of the high-LET induced DSB are not fully understood.

Mammalian cells repair these lesions principally through two separate pathways: homologous recombination (HR), which is thought to rely on the presence of an intact sister chromatid during the S and G2 phases, and nonhomologous end joining (NHEJ), which utilizes DNA repair protein and is thought to predominate in the G1 phase. The NHEJ pathway, however, is regarded as the major pathway for the repair of radiation induced DSB in mammalian cells [4]. Activation of the Ataxia Telangiectasia Mutation (ATM) through its phosphorylation on Ser1981 (ATM–S1981P, pATM), and phosphorylation of one of the variants of histone H2AX, histone H2AX on Ser139 (γH2AX), not only are the main participants, but also the early markers of a cell’s response to DNA damage, particularly if the damage involves formation of DSB [5, 6]. These modifications of ATM and H2AX trigger pathways are involved in DNA repair and in activating checkpoints that halt progression through the cell cycle [7, 8]. The pause in cell cycle progression is needed to allow for DNA repair to succeed prior to resumption of DNA replication or cell division.

High linear energy transfer (LET) radiation, such as heavy ion particles, is believed to produce high yields of clustered DNA damage including DSB [9–11]. A prolonged cell cycle arrest [12] and a slower rejoining of DSB [13] have been reported to occur after exposure to high-LET radiation. However, the repair dynamics of high-LET radiation-induced DNA damage remains poorly understood.

In the present study, the expression of γH2AX and pATM were assay with immunocytochemical and flow cytometry methods and the correlation between clonogenic survival and the level of γH2AX and pATM was evaluated in human cervical carcinoma Hela cells, hepatoma HepG2 cells and mucoepidermoid carcinoma MEC-1 cells after irradiation with ^12^C^6+^. Our studies emphasize the rate of γH2AX and pATM formation and loss may be an important factor in the response of cells to ^12^C^6+^. pATM and γH2AX are effective radiation biomarkers in assessing the radiosensitivity of ^12^C^6+^ in human tumor cells.

## Methods

### Cell lines

Human cervical carcinoma HeLa cell and human hepatoma HepG2 cells were purchased from the Shanghai Institute of Biochemistry and Cell Biology, Shanghai, China. Human mucoepidermoid carcinoma MEC-1 cells were purchased from the School of Stomatology, at the Fourth Military Medical University of Xian, China. The cells were subcultured in Dulbecco’s Modified Eagle Medium (DMEM) (GIBCO, USA), containing 10% newborn calf serum, 100 U/mL penicillin, 125 g/mL streptomycin, and 0.03% glutamine.

### Irradiation using carbon ion beams

Exponentially growing cells seeded at 2 × 10^4^ cells/100 mm dish were exposed to different dosages of ^12^C^6+^. Immediately following irradiation, the medium was quickly removed and cells were incubated for various time intervals at 37 °C before harvest. ^12^C^6+^ was supplied by the Heavy Ion Research Facility in Lanzhou (HIRFL) at the Institute of Modern Physics, Chinese Academy of Sciences (IMP–CAS). Since the energy decays through the vacuum window, air gap, Petri dish cover and medium, the energy of the ion beams on cell samples was adjusted to be 300 meV/u, corresponding to a LET of 15 keV/μm and the dose rate was adjusted to be about 0.4 Gy/min. The ion beams were calibrated using an absolute ionization chamber. The tumor cells were irradiated by plateau of carbon ions LET curve and the dose of scatter off the walls of the plate has been calculated and incorporated into the total dose. The data (preset numbers converted to absorbed dose of particle radiation) was automatically obtained using a microcomputer during irradiation. The dose rate was approximately 1.38 Gy/min and the dose used for ^12^C^6+^ irradiation was 0.5, 1, 2 and 4 Gy.

### Clonogenic survival assays

The cells were cultured for 0.5, 4 and 24 h after irradiation and then were washed with phosphate-buffered saline, trypsinized, and counted using a Coulter counter, replated at a density of 5 × 10^2^–3 × 10^4^ cells in duplicate using 100 mm dishes for cell-survival assays. Plates were stained and colonies were counted two weeks later. Counts from the two plates were averaged, and surviving fraction was calculated as the ratio of the plating efficiency of the treated cells divided by the plating efficiency of the control cells. Experiments were repeated 3–4 times [14]. The survival fraction was calculated using the following formula:

1 $$Survival\, fraction=\frac {\text{No. of colonies}} {\text{No. of cells platting}\times (\text{plating efficiency}/100)}$$

### Immunofluorescence microscopy for γH2AX and pATM foci

Immunofluorescent microscopy was conducted according to previously reported procedures with modifications [15, 16]. Briefly, 2 × 10^4^ cells were seeded onto 35 mm dishes containing a glass cover slip in each well. After irradiation, slides were air-dried, and fixed for 0.5 h in 2% paraformaldehyde in TBS. Cells were rinsed in TBS, placed in −20 °C methanol for 1 min, rinsed, then placed for 20 min in TBS plus 1% bovine serum albumin and 0.2% Tween-20 (TTN) and finally incubated for 2 h with anti-phospho-histone H2AX (Ser-139) mAb (Upstate, Lake Placid, NY), anti-phospho-ATM (ser1981) mAb (Upstate, Lake Placid, NY), both diluted to 1:500 in TTN. Slides were washed and incubated with FITC-conjugated anti-mouse goat F(ab’)^2^ fragment (DAKO, Carpinteria, CA) diluted 1:200 in TTN and FITC-conjugated anti-rabbit goat F(ab’)^2^ fragment (DAKO, Carpinteria,CA) diluted 1:200 in TTN for 1 h at room temperature. Slides were rinsed and then immersed in 0.05 mg/mL DAPI for 15 min, rinsed and mounted with cover slips using 10 μL Fluorogard (Bio-Rad) as the antifade mounting medium, and sealed. To prevent bias in selection of cells that display foci, over 800 randomly selected cells were counted. Cells with three or more foci of any size were classified as positive. All experiments were repeated in triplicate.

### Flow cytometry assay for γH2AX and pATM

Flow cytometry analysis was conducted as previously described [17, 18]. After the various treatments, cells were fixed with cold 70% methanol and kept at −20 °C for up to 2 weeks until further analysis. Cells were centrifuged and rinsed with PBS, blocked with PST (4% fetus bovine serum in PBS) for 15 min at room temperature and rinsed a second time with PBS. Cells were first incubated with Anti-phospho-Histone H2AX (Ser139) mAb (Upstate, Lake Placid, NY) and Anti-phospho-ATM (ser1981) mAb (Upstate, Lake Placid, NY) at 1:300 and 1:100 dilution for 2 h at room temperature, then rinsed with PBS and incubated with Alexa Fluor 488-conjugated AffiniPure Goat Anti-Mouse IgG (H + L) at a 100- and 200-fold dilution for another 1 h at room temperature and rinsed again in PBS. Cells were further incubated for 0.5 h at room temperature with 50 µg/mL PI. Cells were filtered through a 35 μm pore strainer and were analyzed using a flow cytometer (Becton–Dickinson, Bedford, MA, USA). Cell cycle analysis was conducted as described by Amrein et al. [19].

To examine the relationship between the expression of γH2AX and pATM in each phase of the cell cycle, the changes in γH2AX and pATM immunofluorescence intensity (IF) were calculated in each phase of the cycle by gating G1, S and G2/M cells based on differences in DNA content. The means of γH2AX and pATM and positive ratios for G1, S and G2/M populations of cells in the DMSO control groups were subtracted from the respective means of the non-irradiated cells. After this subtraction, the irradiation-induced changes in positive γH2AX and pATM ratio were obtained. Data is presented as the mean of the γH2AX and pATM positive ratios of each cell cycle compartment. All experiments were performed three times.

### Statistical analysis

SPSS version 18.0 software (SPSS Inc., Chicago, Illinois, USA) was used for the statistical analysis. Data are expressed as mean ± standard deviation (SD). A two-tailed Student’s t test was performed to assess the differences between any two groups. The significance of the correlation coefficient was also calculated. A value of P < 0.05 was considered statistically significant. Statistical inferences were based on two-sided tests at a significance level of P < 0.05.

## Results

### Growth dynamics of colony survival assay

**Fig. 1:**
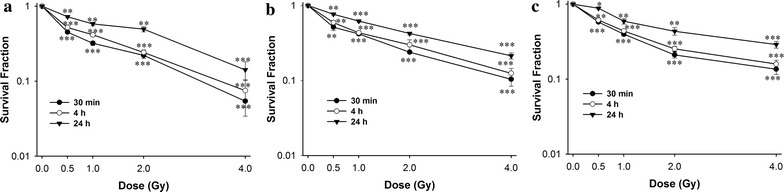
A survival curve for the Hela, HepG2 and MEC-1 cell lines, as determined by clonogenic assay. Exponentially growing cells were plated and irradiated, the cells were taken at the indicated time intervals after irradiation of ^12^C^6+^ and a clonogenic assay was performed. The means and SD are shown for three independent experiments with 3 replicates in each experiment. Untreated cells served as a control. After incubation for two weeks, colonies with cells greater than 50 were counted. **a** Hela cells; **b** HepG2 cells; **c** MEC-1 cells. *P < 0.05, **P < 0.01, ***P < 0.001 vs. 0 Gy irradiation

Clonogenics cells were inactivated immediately, but in turn, significantly increased during 24 h after ^12^C^6+^ irradiation (P < 0.05). The survival fraction decreased in dose-dependent manner at every time point for each tumor cells (P < 0.05, Fig. 1).

### Immunofluorescence staining of phosphorylated H2AX and ATM foci

**Fig. 2:**
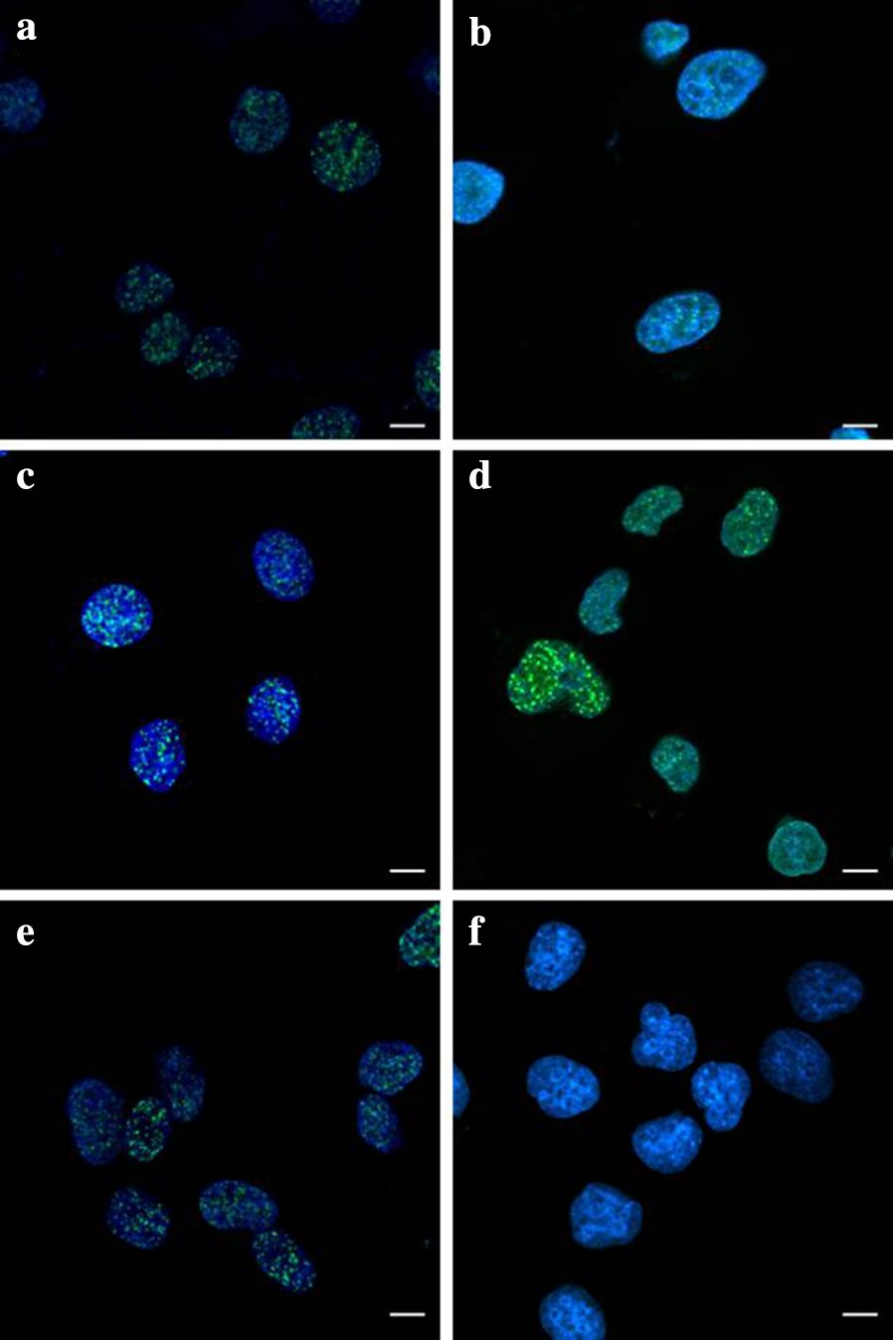
Digitized images of γH2AX and pATM foci. After exposure to 2 Gy ^12^C^6+^ and incubation 0.5 h for γH2AX and 4 h for pATM, cells were grown and irradiated on cover slips. DNA was stained with DAPI and γH2AX and pATM was detected using an Alexa 488-conjugated secondary antibody after staining using anti-phospho-histone H2AX (Ser-139) and anti-phospho-ATM (ser1981) mAb. **a** Hela-γH2AX; **b** Hela-pATM; **c** HepG2-γH2AX; **d** HepG2- pATM; **e** MEC-1-γH2AX; **f** MEC-1-pATM. *Scale bar* 15 μm

**Fig. 3:**
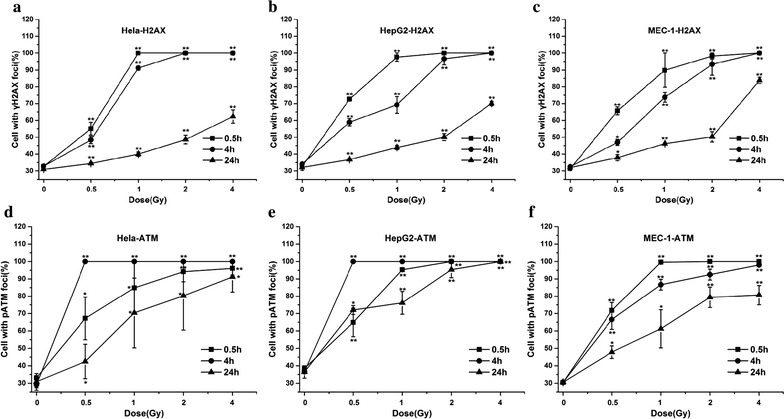
Foci formation of γH2AX and pATM in Hela, HepG2 and MEC-1 cells observed by immunofluorescent microscopy. The three cell lines are exposured to 0.5, 1, 2 and 4 Gy ^12^C^6+^ and subsequently incubated for 0.5, 4 and 24 h for γH2AX and pATM in vitro. **a**, **b**, **c** γH2AX; **d**, **e**, **f** pATM; **a**, **d** Hela cells; **b**, **e** HepG2 cells; **c**, **f** MEC-1 cells. *P < 0.05 vs. 0 Gy irradiation; **P < 0.01 vs. 0 Gy irradiation. Over 800 randomly selected cells were counted. Cells with three or more foci of any size were classified as positive. Results are the means and SD for the three experiments

Phosphorylated H2AX and ATM foci were observed with anti-γH2AX antibodies (green), anti- ATMpSer1981 antibodies (green) and the nuclei were stained with DAPI (blue). Typical images of ^12^C^6+^ induced γH2AX and pATM foci are shown in Fig. 2. After 0.5 h of radiation, γH2AX and pATM foci, visualized as bright spots, were present in all cells. The time and dose dependent induction of γH2AX and pATM foci by ^12^C^6+^ were counted in all tumor cell lines. It was noted that the strongest inductions of γH2AX foci were at 0.5 h for all three tumor cell lines. However, the strongest induction of pATM foci was at 4 h for HeLa and HepG2 cells and at 0.5 h for MEC-1 cells, and then decreased over time. A fraction foci persisted for at least 24 h for γH2AX and pATM for all three tumor cells, for example, about 62.2–83.8% γH2AX foci and 80.7–100% pATM foci were shown in three cell lines after 4 Gy radiation (Fig. 3).

### ^12^C^6+^ induces H2AX and ATM phosphorylation in a cell cycle-dependent manner

**Fig. 4:**
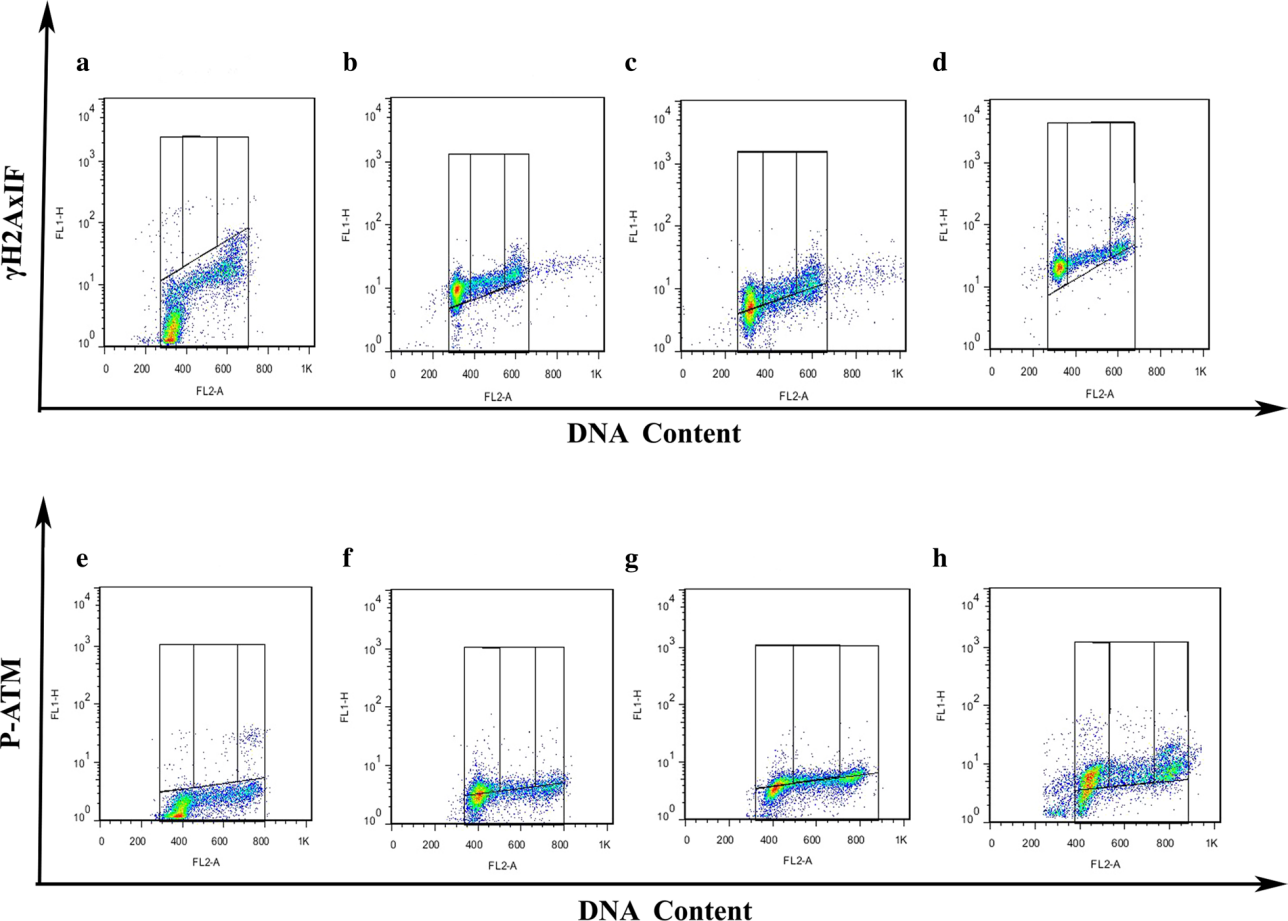
γH2AX and pATM in a cell cycle-dependent manner in Hela, HepG2 and MEC-1 cells. Bivariate (γH2AX and pATM IF vs DNA content) distributions of control and 4 Gy ^12^C^6+^ irradiation and subsequent incubation for 0.5 h for γH2AX and 4 h for phosphorylated ATM in vitro. **a**, **b**, **c**, **d** γH2AX; **e**, **f**, **g**, **h** pATM; **a**, **e** Control (Hela cells); **b**, **f** Hela cells; C,G-HepG2 cells; **d**, **h** MEC-1 cells

In order to further determine the phosphorylation levels of H2AX and ATM, the intensity of γH2AX and pATM were assayed with flow cytometry. Typical flow cytometry histograms of ^12^C^6+^ induced phosphorylation of H2AX and ATM in a cell cycle-dependent manner are shown in Fig. 4.

**Fig. 5:**
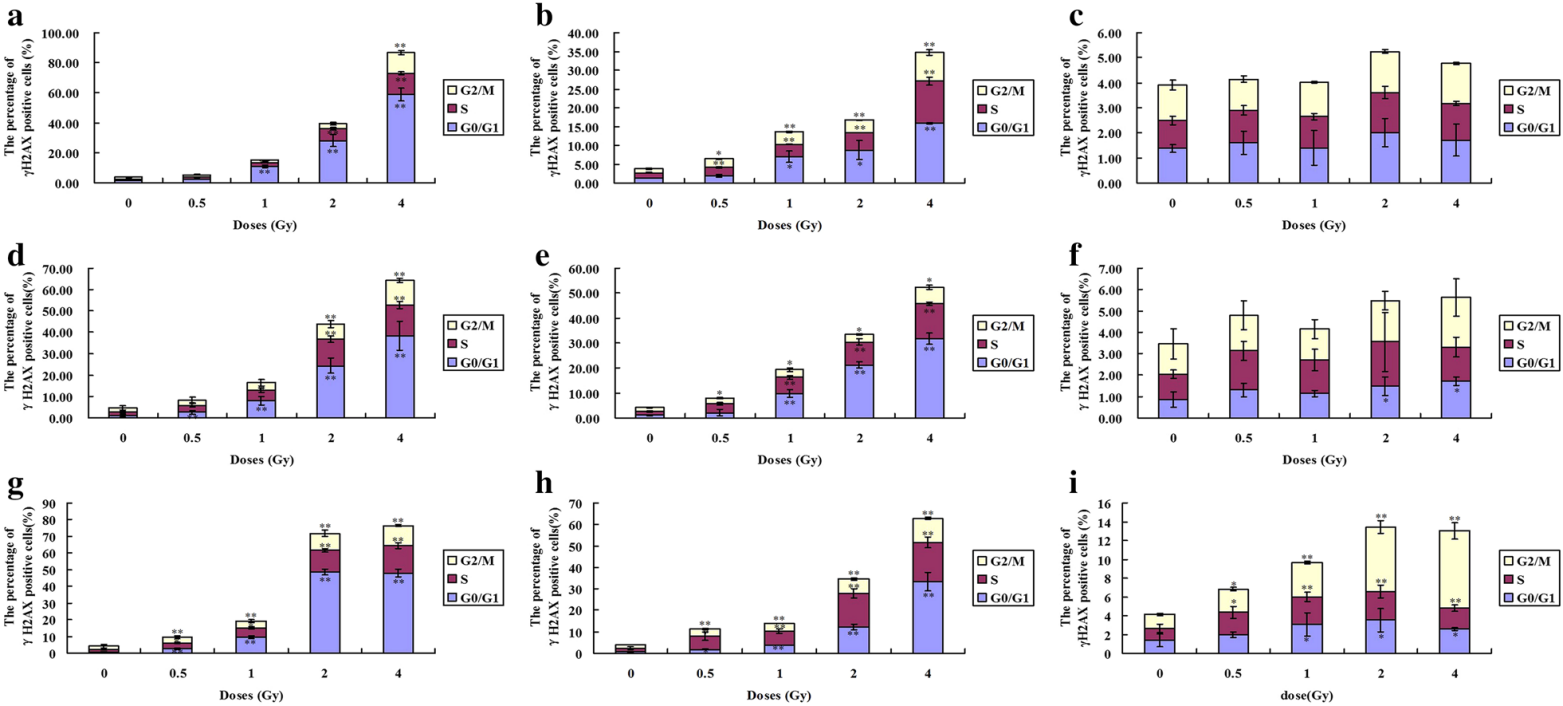
The expression of γH2AX in a cell cycle-dependent manner in Hela, HepG2 and MEC-1 cells. The three cell lines are exposed to 0.5, 1, 2 and 4 Gy ^12^C^6+^ irradiation and then incubated for 0.5, 4 and 24 h in vitro. **a**, **b**, **c** Hela cells; **d**, **e**, **f** HepG2 cells; **g**, **h**, **i** MEC-1cells; **a**, **d** G-0.5 h; **b**, **e**, **h** 4 h; **c**, **f**, **i** 24 h. *P < 0.05, **P < 0.01 vs Control. Results are the means and SD for the three experiments

**Fig. 6:**
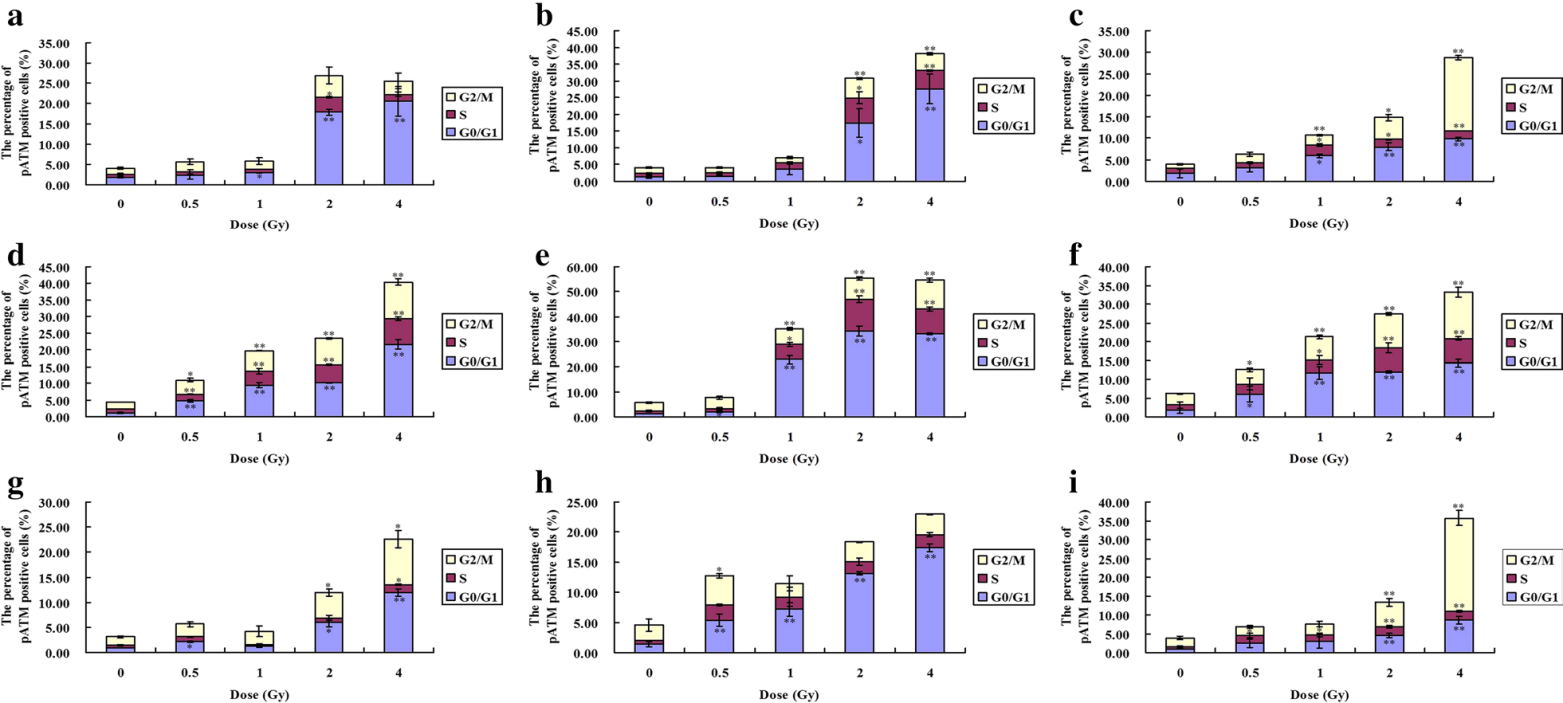
The expression of pATM in a cell cycle-dependent manner in Hela, HepG2 and MEC-1 cells. The three cell lines are exposed to 0.5, 1, 2 and 4 Gy ^12^C^6+^ irradiation then incubated for 0.5, 4 and 24 h in vitro. **a**, **b**, **c** Hela cells; **d**, **e**, **f** HepG2 cells; **g**, **h**, **i** MEC-1cells; **a**, **d** G-0.5 h; **b**, **e**, **h** 4 h; **c**, **f**, **i** 24 h. *P < 0.05, **P < 0.01 vs Control. Results are the means and SD for the three experiments

After 0.5 and 4 h irradiation, the percentage of γH2AX positive cells increased in a dose dependent manner in almost all phases, in which, G0/G1 phase cells had the highest expression of γH2AX after 0.5 h irradiation and then decreased to a lower level at 24 h after irradiation (Fig. 5). An obvious increase of pATM in G2/M was shown after 24 h of 2 and 4 Gy irradiation (Fig. 6).

**Fig. 7:**
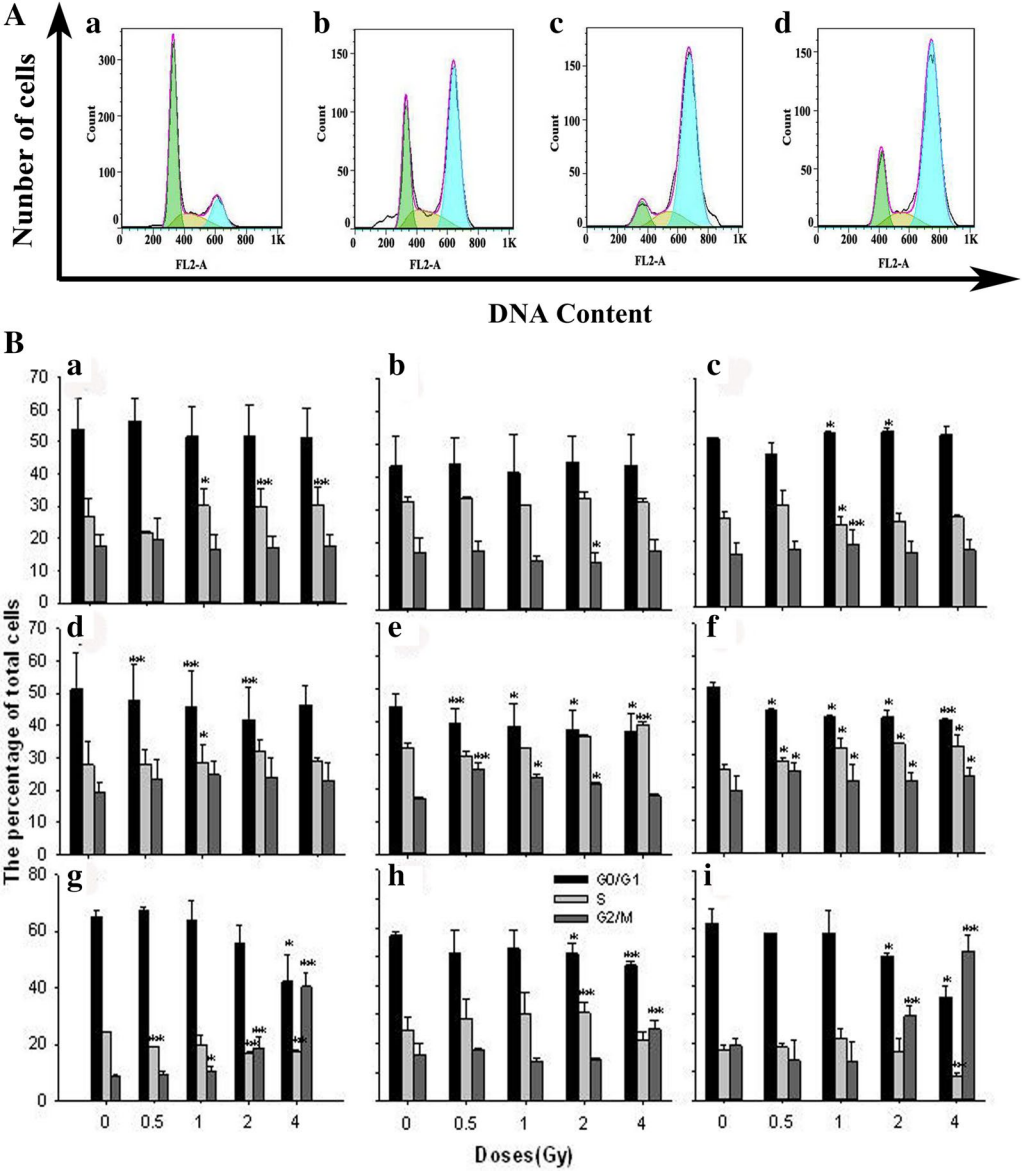
**A** Cell cycle distribution of three cell lines. *a*-Hela, HepG2 and MEC-1 cell are treated with 4 Gy ^12^C^6+^ followed incubation for 24 h. *a* Control (Hela cells), *b* Hela cells, *c* HepG2 cells, *d* MEC-1 cells. **B** Three cell lines treated with 0.5, 1, 2 and 4 Gy ^12^C^6+^ irradiation and subsequently incubated for 0.5, 4 and 24 h. *a, b*, *c* 0.5 h;* d*, *e*, *f* 4 h; *g*, *h*, *i* 24 h. *a*, *d*, *g* Hela cells; *b*, *e*, *h* HepG2 cells; *c*, *f*, *i* MEC-1 cells. *P < 0.05, **P < 0.01 vs Control. Results are the means and SD for the three experiments

The effect of the cell cycle of the three tumor cell lines for ^12^C^6+^ exposure is presented in Fig. 7. There was a significant G2/M phase arrest. For example, after 4 Gy irradiation, there were 40.5% Hela cells in G2/M after 24 h vs. 17.8% in G2/M after 0.5 h and there were about 25.0 and 51.9% of HepG2 and MEC-1 cells in G2/M after 24 h vs, 17.9 and 17.6% in G2/M after 0.5 h.

### The correlation between the clonogenic survival and γH2AX and pATM foci in ^12^C^6+^ irradiated tumor cells

In order to determine if there was a direct quantitative relationship between the clonogenic survival and γH2AX and pATM expression, cells were exposed to different dose ^12^C^6+^ and incubated to different time. A positive correlation was shown between the clonogenic survival and γH2AX and pATM foci. The correlation coefficients for almost all parameters we used, such as different doses and irradiated time points, were statistically significant (P < 0.05, Tables 1, 2) suggesting that these two variables are directly linked.

**Table 1:** Correlation coefficient obtained from γH2AX by correlating expression with the SF

	0.5 h		4 h		24 h	
	r values	P values	r values	P values	r values	P values
Hela	−0.91	0.03	−0.906	0.03	−0.964	<0.01
HepG2	−0.954	0.01	−0.978	<0.01	−0.955	0.01
MEC-1	−0.988	<0.01	−0.969	0.01	−0.879	0.05

**Table 2:** Correlation coefficient obtained from pATM by correlating expression with the SF

	0.5 h		4 h		2 4 h	
	r values	P values	r values	P values	r values	P values
Hela	−0.984	<0.01	−0.875	0.05	−0.943	0.02
HepG2	−0.944	0.02	−0.856	0.06	−0.955	0.01
MEC-1	−0.962	0.01	−0.986	<0.01	−0.976	<0.01

## Discussion

In the present study, radiosensitivities of different tumour cell lines to ^12^C^6+^ were established using the clonogenic assay. We selected three tumor cell lines which were of different tissue origins. The different cell types were used to ensure that the assay was able to distinguish the radiosensitivity across different tumor types. In the clonogenic assay, a significantly survival inhibition was shown in ^12^C^6+^ irradiation over time and dose (Fig. 1). It, therefore, seemed reasonable to conclude that an early significant increase in the survival fraction within 24 h occurred after ^12^C^6+^ irradiation.

A cytological manifestation of nuclear activity in response to ionizing radiation (IR) is the formation of the so-called IR-induced foci (IRIF) [20]. IRIFs are dynamic, microscopically discernible structures containing thousands of copies of proteins, including γH2AX, ATM, CHK2, p53 and MRE11/RAD50/NBS1 (MRN) complex, which accumulate in the vicinity of a DSB [21, 22]. Phosphorylation of histone H2AX is among the earliest changes to occur at sites of DSB damage, where it is thought to facilitate repair through maintaining structural changes in chromatin. γH2AX induction following exposure to IR is reported to be mediated by ATM and DNA-PK [23]. The phosphorylation of H2AX by ATM occurs at sites of DSB in the cell nucleus whereas ATM autophosphorylation is thought to take place throughout the nucleoplasm. The figures shown here provide a visualization of ^12^C^6+^ ion tracks inside nuclei in human cells by utilizing immunocytochemical methods with antibodies recognizing γH2AX and pATM (Fig. 2). This assay is quite sensitive and is a specific indicator for the existence of a DSB [24–26].

In the present study, we firstly compared the background values of γH2AX and pATM in three tumor cell lines. The expression of endogenous γH2AX and pATM foci was lower and there was not a significant difference between the three tumor cell lines we used (P > 0.05). We, then, measured foci frequency for up to 24 h and found that a fraction of foci persisted for at least 24 h after high LET carbon ions radiation (Fig. 3). This confirms the earlier studies that these persistent γH2AX and pATM foci as evidence of persistent DSB.

Then we confirmed the induction of DSB as measured by γH2AX and pATM signaling in three cell lines occurs in a dose-dependent manner, as expected, but that foci formation and resolution is different (Fig. 3). The highest level of γH2AX and pATM foci presence in ^12^C^6+^ irradiated cells at 0.5 h or 4 h after irradiation indicates the repair of damage began early in tumor cells. γH2AX foci resolution in MEC-1 cells were seemly delayed and incomplete compared to the other two cell lines because MEC-1 cells expressed higher levels of γH2AX foci even 24 h after 4 Gy irradiation. HepG2 cells had the highest levels of pATM foci at 24 h after 2 and 4 Gy irradiation, so pATM foci resolution in HepG2 cells is also delayed and incomplete compared to other two cells. The data presented here suggest that, presumably as a result of loss of function in some aspects of DNA repair, MEC-1 cells are slowest to repair and are left with more residual damage than the other two tumor cells, as measured by γH2AX foci resolution. When measured by pATM foci resolution HepG2 cells are also slower to repair than the other two cell lines. Of course, foci resolution is not an exact measurement of repair kinetics; recent data suggest that dephosphorylation of H2AX occurs with a significant lag after DSB repair, following protein dissociation from chromatin. Interestingly, this dephosphorylation event may promote checkpoint recovery [27]. In a word, in the present study the higher activation of ATM shown at 4 h compared with H2AX phosphorylation at 0.5 h and γH2AX and pATM foci delayed resolution in MEC-1 and HepG2 cells maybe highlight signaling differences with respect to clustered damage. Concurrent activation of ATM and γH2AX suggest that the latter event, at least in part, was independent in ATM.

In order to further determine the phosphorylation levels of H2AX and ATM, the intensity of γH2AX and pATM were assayed with flow cytometry. Our result proved the expression of γH2AX and pATM was in relation to cell cycle. Flow cytometry, which is a convenient method for detecting differences in γH2AX and pATM antibody binding in populations of cells, offers the advantage of measuring change in γH2AX and pATM intensity in relation to the cell cycle position [28–30]. Olive PL assessed the expression of γH2AX phosphorylation by flow cytometry to detect and measure DNA damage induced by X-rays. It has also been reported that cytometric assessment of γH2AX fluorescence decay in blood cells of X-irradiated patients and low and high LET radiated cells offers a sensitive measure of DNA damage in vivo and in vitro [31]. Flow cytometry also offers the advantage of measuring changes in phosphorylated ATM intensity in relation to cell cycle position in mitogenic stimulated lymphocytes and glucose antimetabolite 2-deoxy-d-glucose (2-DG) treated B-lymphoblastoid TK6 cells [32, 33]. Analysis of the post-irradiation kinetics of γH2AX and pATM fluorescence with flow cytometry revealed a pattern which suggests that G0/G1,S and G2/M phase cells vary independently on the relative expression of γH2AX and pATM. The present study demonstrates that G0/G1 phase cells are more uniformly affected than S and G2/M phase cells. For example, at 0.5 h after 4 Gy ^12^C^6+^ irradiation, over 40% of G0/G1 phase cells had increased expression of γH2AX and a little decrease was shown at 4 h after irradiation in all three cell lines (Fig. 5). Although G0/G1 phase cells had increased expression of pATM, interesting, the G2/M cells showed a significantly increase of pATM at 24 h after 2 and 4 Gy ^12^C^6+^ irradiation (Fig. 6).

Tumor cells used here show no significant G1 checkpoint response after irradiation [33]. However, the data presented here demonstrates a clear dose response G2 checkpoint that prolongs the G2 phase by several hours even after very low radiation doses (Fig. 7). These data imply that the tumor cells used here are relatively more dependent on the G2 checkpoint to facilitate repair. Others have recently described this phenotype in other tumor cell lines and demonstrated that it predicts sensitivity to G2 checkpoint inhibition [34].

The results of the γH2AX and pATM expression were compared with those of clonogenic assay in determining the radiosensitivity of the tumour cell lines. For the three cell lines, the DNA repair kinetics after ^12^C^6+^ irradiation, as measured using γH2AX and pATM foci assay, were strongly correlated with the radiosensitivity of clonogenicity, which is in agreement with our former report [14], in which we proved γH2AX foci assay had the potential value in assessing the radiosensitivity of carbon beam in human tumor cell lines.

## Conclusion

Our result suggests the rate of γH2AX and pATM formation and loss may be an important factor in the response of cells to ^12^C^6+^. pATM and γH2AX are effective radiation biomarkers in assessing the radiosensitivity of ^12^C^6+^ in human tumor cells.

## Abbreviations

DMEM: Dulbecco’s modified Eagle’s medium
PI: propidium iodide
DMSO: dimethyl sulfoxide
γH2AX: H2AX phosphorylation
ATM: Ataxia telangiectasia-mutated
DNA-PK: DNA dependent protein kinase
MRN: MRE11/RAD50/NBS
DSB: DNA double strand breaks
NHEJ: non-homologous end-joining
HR: homologous recombination
IRIF: IR-induced foci
